# TAS4464, a NEDD8-activating enzyme inhibitor, activates both intrinsic and extrinsic apoptotic pathways via c-Myc-mediated regulation in acute myeloid leukemia

**DOI:** 10.1038/s41388-020-01586-4

**Published:** 2021-01-08

**Authors:** Hiroaki Ochiiwa, Guzhanuer Ailiken, Masataka Yokoyama, Kazuyuki Yamagata, Hidekazu Nagano, Chihoko Yoshimura, Hiromi Muraoka, Keiji Ishida, Tomonori Haruma, Akitoshi Nakayama, Naoko Hashimoto, Kazutaka Murata, Motoi Nishimura, Yusuke Kawashima, Osamu Ohara, Shuichi Ohkubo, Tomoaki Tanaka

**Affiliations:** 1 Discovery and Preclinical Research Division, Taiho Pharmaceutical Co., Ltd, 3 Okubo, Tsukuba, Ibaraki 300-2611 Japan; 2 Department of Molecular Diagnosis, Graduate School of Medicine, Chiba University, Chiba, 260-8670 Japan; 3 Division of Laboratory Medicine, Clinical Genetics and Proteomics, Chiba University Hospital, Chiba, 260-8670 Japan; 4 Department of Applied Genomics, Kazusa DNA Research Institute, Kisarazu, Chiba 292-0818 Japan

**Keywords:** Haematological cancer, Apoptosis

## Abstract

TAS4464, a potent, selective small molecule NEDD8-activating enzyme (NAE) inhibitor, leads to inactivation of cullin-RING E3 ubiquitin ligases (CRLs) and consequent accumulations of its substrate proteins. Here, we investigated the antitumor properties and action mechanism of TAS4464 in acute myeloid leukemia (AML). TAS4464 induced apoptotic cell death in various AML cell lines. TAS4464 treatments resulted in the activation of both the caspase-9-mediated intrinsic apoptotic pathway and caspase-8-mediated extrinsic apoptotic pathway in AML cells; combined treatment with inhibitors of these caspases markedly diminished TAS4464-induced apoptosis. In each apoptotic pathway, TAS4464 induced the mRNA transcription of the intrinsic proapoptotic factor NOXA and decreased that of the extrinsic antiapoptotic factor c-FLIP. RNA-sequencing analysis showed that the signaling pathway of the CRL substrate c-Myc was enriched after TAS4464 treatment. Chromatin immunoprecipitation (ChIP) assay revealed that TAS4464-induced c-Myc bound to the *PMAIP1* (encoding NOXA) and *CFLAR* (encoding c-FLIP) promoter regions, and siRNA-mediated c-Myc knockdown neutralized both TAS4464-mediated NOXA induction and c-FLIP downregulation. TAS4464 activated both caspase-8 and caspase-9 along with an increase in NOXA and a decrease in c-FLIP, resulting in complete tumor remission in a human AML xenograft model. These findings suggest that NAE inhibition leads to anti-AML activity via a novel c-Myc-dependent apoptosis induction mechanism.

## Introduction

Acute myeloid leukemia (AML) is a heterogeneous disease characterized by numerous genetic mutations and chromosomal abnormalities [1]. Although extensive efforts are being made to develop novel therapeutics, current chemotherapies have shown limited efficacy and overall survival has not been significantly changed because most AML patients develop chemoresistance during treatment and relapse after exhibiting an initial response [2]. Recently, the expression level of antiapoptotic factors has been widely implicated in chemoresistance and correlated with poor prognosis in AML [3–6]. Therefore, modulation of apoptosis-related proteins has emerged as a promising treatment strategy. Indeed, a novel BCL-2 inhibitor, venetoclax, was recently approved for use in combination with decitabine, azacitidine, or low-dose cytarabine for the treatment of adult patients with AML [7]. However, as in the case of essentially all targeted agents, intrinsic or acquired resistance to this agent generally occurs, prompting us to develop new apoptosis regulators to treat AML.

Neddylation is one of the posttranscriptional modification processes by which NEDD8 is conjugated to substrate proteins [8]. NEDD8 is a ubiquitin-like protein that can induce a conformational change in its target substrates, and neddylation is thought to control various biological processes including cell proliferation, cellular senescence, and apoptosis [9–13]. Conjugation of NEDD8 to its substrates is catalyzed by NEDD8-activating enzyme E1 (NAE; a heterodimer comprising the APPBP1 and UBA3 subunits) and NEDD8-conjugating enzyme E2 (Ubc12/UBE2M or UBE2F) [14, 15]. The most well-characterized neddylation substrates are cullin family proteins, which are core scaffold components of cullin-RING E3 ubiquitin ligases (CRLs) [16, 17]. The CRLs family is the largest ubiquitin E3 ligase family, and its members are activated by neddylation. Activated CRLs promote to the conjugation of ubiquitin to their substrate proteins, and ubiquitinated proteins are degraded via the ubiquitin-proteasome system [18]. To date, several studies have reported that neddylation is involved in tumor growth and survival [19–21]. Therefore, inhibition of the neddylation pathway by targeting NAE is considered as a novel therapeutic approach for cancers to disrupt cell growth or survival. Indeed, MLN4924/TAK-924 (pevonedistat), an NAE inhibitor, has been developed as a clinical antitumor agent [22–26].

We recently reported a novel NAE inhibitor, TAS4464, which is the most potent and selective NAE inhibitor reported to date and shows widespread antiproliferative activity against various cancer cell lines [27]. TAS4464 also had a broad therapeutic index due to its long-acting NAE inhibition effect in tumors and reduced amount of off-target effects. Although MLN4924 has been reported to induce apoptosis in AML cells through induction of the proapoptotic BCL-2 family protein NOXA [28], the molecular mechanism underlying NOXA induction has not been fully elucidated. Here, we extensively investigated the effect of TAS4464 in AML cells and found that TAS4464 activates both the intrinsic and extrinsic apoptotic pathways. TAS4464 activates caspases by increasing the NOXA level and decreasing the c-FLIP level. These molecules are transcriptionally regulated by c-Myc as direct targets. Indeed, we confirmed the tumor remission with TAS4464 treatment in a human AML xenograft model after the involvement of target molecules. This TAS4464-mediated antitumor mechanism could explain how NAE inhibition can be applied in patients as a novel therapeutic strategy.

## Results

### TAS4464 induces apoptosis in AML cell lines independent of their genetic background

**Fig. 1:**
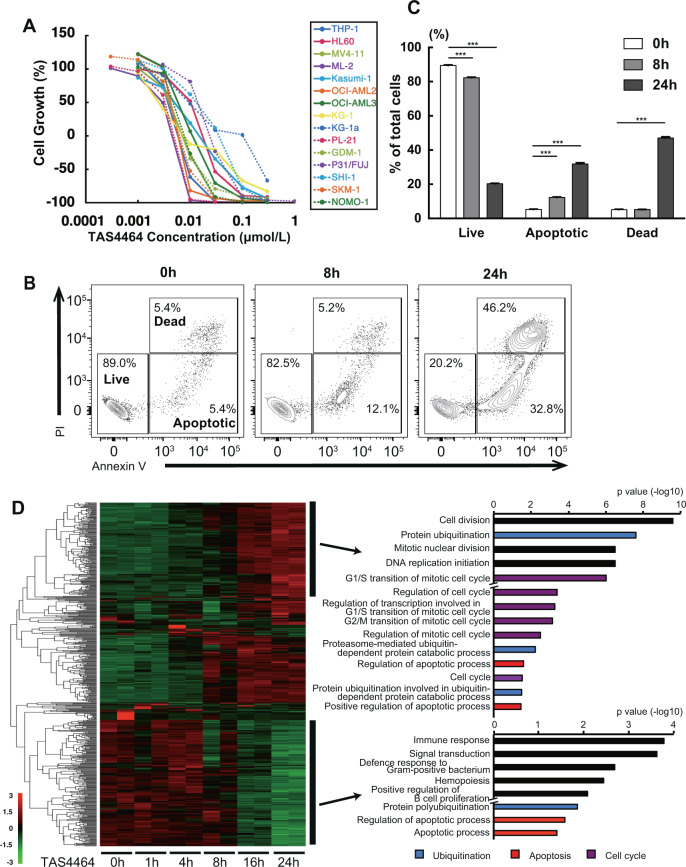
TAS4464 induces apoptosis in AML cell lines. **A** AML cell lines were seeded in 96-well plates and treated the next day with various concentrations of TAS4464. After 3 days, cell viability was determined by measurement of the cellular ATP contents. **B** Apoptotic cell death was evaluated by flow cytometric analysis. HL-60 cells were treated with 0.1 μmol L^−1^ TAS4464 for 0, 8, and 24 h. **C** Total percentage of cells with each status shown in (**B**). **D** Heatmap showing differentially expressed molecules identified by proteomics analysis in HL-60 cells. Cells were treated with TAS4464 (0.1 μmol L^−1^) for 0, 1, 4, 8, 16, and 24 h (left). Pathways enriched with time-dependently upregulated or downregulated molecules, as identified by Gene Ontology analysis, are listed (right).

To evaluate the tumor-suppressive effect of TAS4464, we measured the ATP content in AML cell lines after treatments. Although each tested AML cell line harbored various types of genetic abnormalities such as *MLL-AF9* or *FLT3-ITD* translocation, TAS4464 treatments decreased cell viability, which was accompanied by cell death in all evaluated AML cell lines (Fig. 1A).

To address the mechanism of TAS4464-induced tumor suppression, cell-cycle profiles were analyzed by flow cytometry in two AML cell lines, HL-60 and THP-1 cells, by treatment with 0.1 μmol L^−1^ TAS4464 treatment. Among the cell-cycle phases investigated, including the G1/S and G2/M transition, cells of both lines were found to accumulate in sub-G1 phase over time after up to 24 h of TAS4464 exposure (Supplementary Fig. S1a, b), suggesting that TAS4464 induced apoptosis in AML cell lines independent of their genetic background. Then, we confirmed by Annexin V staining that the apoptotic cell death was induced by TAS4464 treatment in HL-60 cells (Fig. 1B, C). Next, we performed proteomics analysis and selected the differentially expressed molecules in HL-60 cells at time points of up to 24 h after TAS4464 treatment to evaluate the overall protein expression profile (Fig. 1D). Gene Ontology analysis revealed that TAS4464 treatment influenced apoptotic pathways, cell cycle-related pathways and ubiquitination pathways over time (Fig. 1D). Considering these results collectively, we assumed that TAS4464 treatment induces apoptosis in AML cells and inhibits tumor growth as an underlying mechanism accompanied by degradation pathway activity.

### TAS4464 activates both the intrinsic and extrinsic apoptotic pathways

**Fig. 2:**
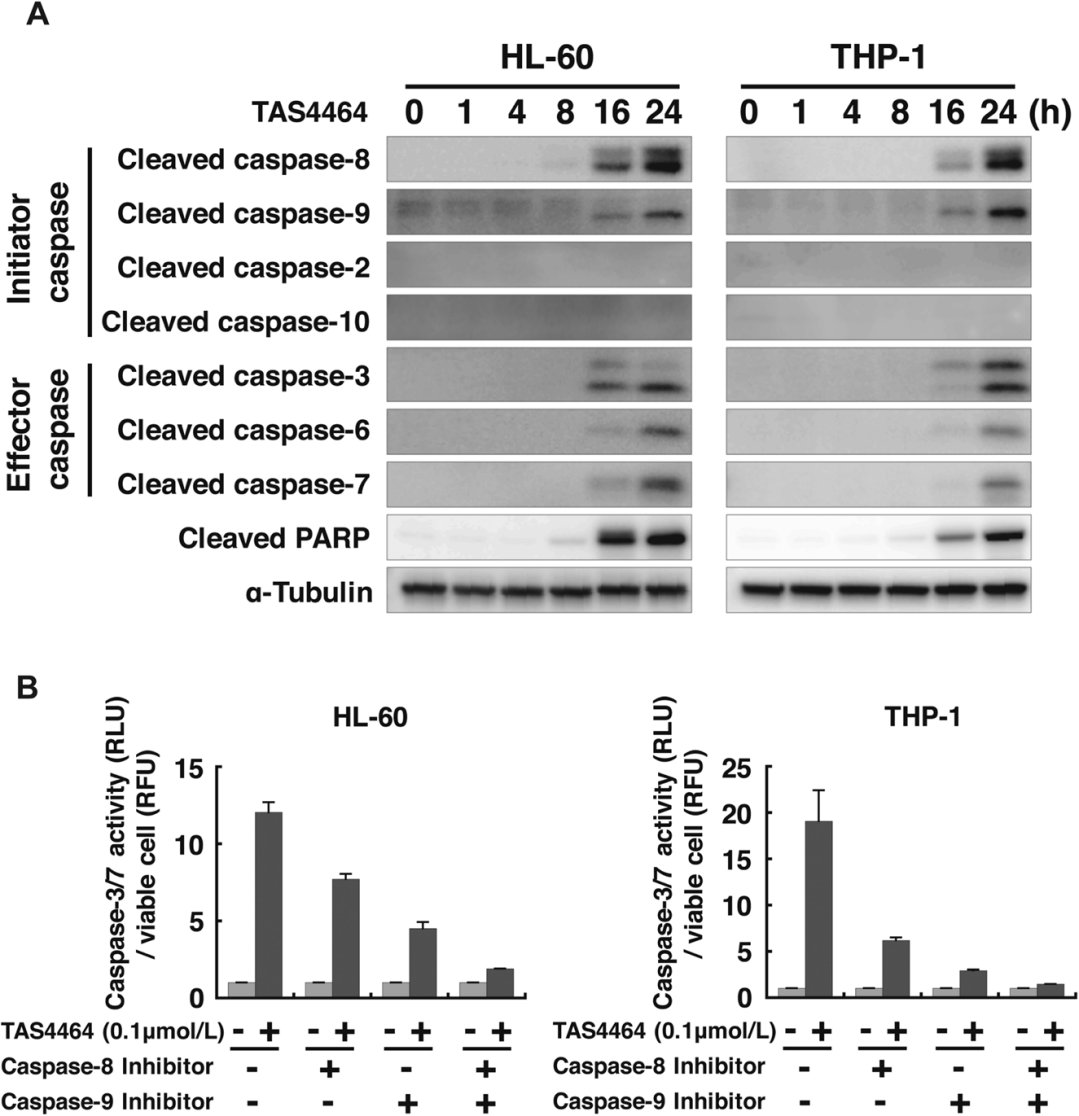
TAS4464 activates both intrinsic and extrinsic apoptotic pathways. **A** HL-60 and THP-1 cells were treated with 0.1 μmol L^−1^ TAS4464 for 1, 4, 8, 16, and 24 h, and total protein was extracted. The active form of each caspase was detected with the indicated antibodies. **B** Cells were treated with 1 μmol L^−1^ z-IETD-FMK alone, 1 μmol L^−1^ z-LEHD-FMK alone, and a combination of these inhibitors for 1 h prior to exposure to TAS4464. Then, cells were treated with 0.1 μmol L^−1^ TAS4464 for 16 h. Caspase-3/7 activity levels are expressed as relative luminescence units (RLU), which were normalized to the number of viable cells under each condition (relative fluorescence units, RFU). Data are presented as the mean ± SEM values of data from three independent experiments.

There are two well-characterized apoptosis cascades: the extrinsic and intrinsic pathways, which are mediated by caspase-8 and caspase-9, respectively [29]. The other caspase family members play critical roles as initiator or effector caspases in each apoptotic pathway [30]. To assess the involvement of these apoptotic pathways in TAS4464-induced tumor suppression, changes in the caspase activities upon treatment with TAS4464 were evaluated in HL-60 and THP-1 cells. TAS4464 treatment activated caspase-8 and caspase-9 among the initiator caspases (Fig. 2A), suggesting that TAS4464 activates both the intrinsic and extrinsic apoptotic pathways.

To examine whether these initiator caspases are involved in TAS4464-induced apoptosis, the cells were pretreated with caspase inhibitors—either z-IETD-FMK for caspase-8 or z-LEHD-FMK for caspase-9—for 1 h and were then treated with 0.1 μmol L^−1^ of TAS4464 for an additional 16 h. Although TAS4464 alone activated the downstream effector caspases caspase-3 and caspase-7 in the cells, both of the caspase inhibitors suppressed the activation of those caspases (Fig. 2B). In particular, combined treatment with the caspase-8 and caspase-9 inhibitors markedly diminished TAS4464-induced activation of caspase-3 and caspase-7. These results indicate that not only the intrinsic apoptotic pathway mediated by caspase-9 but also the extrinsic pathway mediated by caspase-8 is also involved in TAS4464-induced apoptosis in AML cells.

### TAS4464 increases NOXA expression and decreases c-FLIP expression at the mRNA transcriptional level

**Fig. 3:**
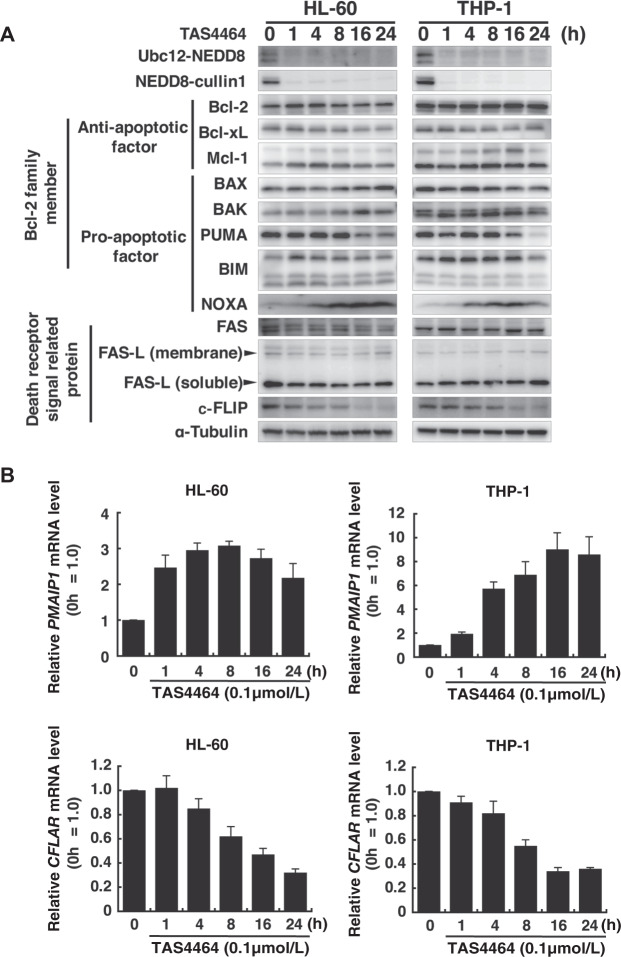
TAS4464 increases NOXA expression and decreases c-FLIP expression at the mRNA transcriptional level. **A** HL-60 and THP-1 cells were treated with 0.1 μmol L^−1^ TAS4464 for 1, 4, 8, 16. and 24 h, and total protein was extracted. Immunoblotting for neddylated Ubc12 and cullin1 was performed with the anti-NEDD8 antibody. Parallelly, immunoblotting for Bcl-2 family proteins in the intrinsic apoptotic pathway and death receptor signal-related proteins in the extrinsic apoptotic pathway was performed with the indicated antibodies. **B** qRT-PCR was performed to compare the mRNA levels of *PMAIP1* and *CFLAR* in cells treated with 0.1 μmol L^−1^ TAS4464 and control cells (0 h) at the indicated time points (hours). *PMAIP1* and *CFLAR* mRNA expression levels were normalized to 18S rRNA expression levels. Data are presented as the mean ± SEM values from three independent experiments.

We previously reported the inhibitory effect of TAS4464 on the neddylation pathway in several hematopoietic cancers [27, 31], and in this study, a similar drug functionality of TAS4464 was shown in AML cell lines by confirming the decrease in NEDD8 conjugation by Ubc12 or cullin1 (Fig. 3A). To identify the apoptotic factors involved in TAS4464-mediated apoptosis as upstream signaling mediators of caspase activity, we surveyed Bcl-2 family proteins and death receptor signal-related proteins. Protein analysis showed that TAS4464 treatment time-dependently increased the expression of NOXA among the Bcl-2 family proteins and reduced that of c-FLIP among the death receptor signal-related proteins in HL-60 and THP-1 cells (Fig. 3A). c-FLIP, an antiapoptotic protein, can bind to the FADD in competition with caspase-8, resulting in inhibition of death receptor-mediated apoptosis [32]. This drug effect was also observed in the increase in the mRNA levels of *PMAIP1* (the gene encoding NOXA) and in the decrease in that of *CFLAR* (the gene encoding c-FLIP) by Quantitative reverse-transcribed polymerase chain reaction (qRT-PCR) analysis (Fig. 3B), suggesting that TAS4464 modulates these molecules at the transcriptional level.

### TAS4464 treatment activates c-Myc pathway by escaping protein degradation

**Fig. 4:**
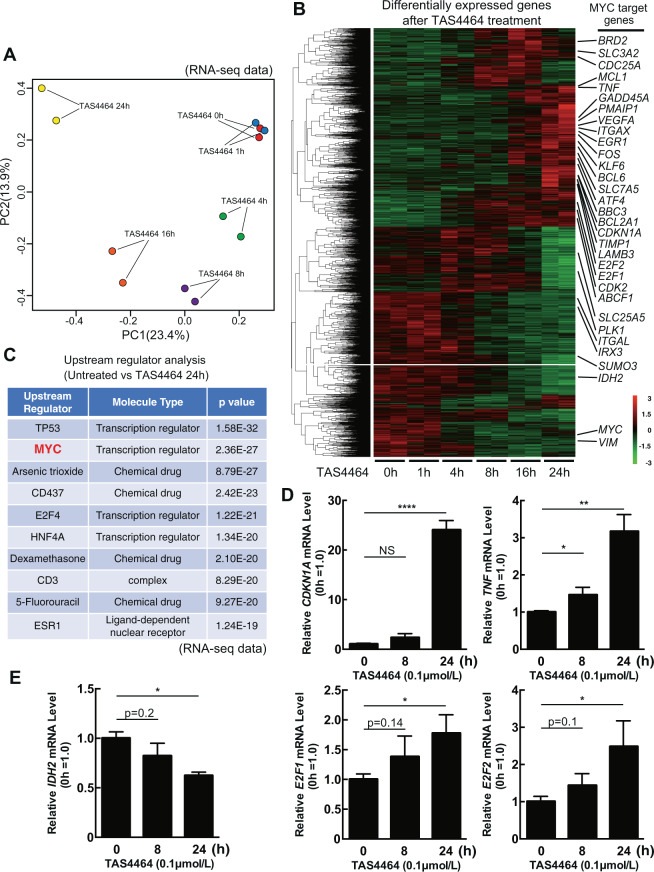
RNA-seq results for TAS4464-treated HL-60 cells. **A** Principal component analysis showing the clusters of HL-60 cells depending on the duration of TAS4464 treatment. DMSO (control) was used for the untreated condition. **B** RNA-seq data for each population. Differentially expressed mRNAs are listed using hierarchical clustering. Representative c-Myc target genes are shown in the list. **C** Upstream regulators were predicted using Ingenuity Pathway Analysis based on the difference in gene expression between untreated cells and cells treated with TAS4464 for 24 h. qRT-PCR was performed to compare the mRNA levels of *CDKN1A, TNF, E2F1, E2F2* (**D**) and *IDH2* (**E**) in cells treated with 0.1 μmol L^−1^ TAS4464 and control cells. 18S rRNA expression was used for normalization and data are presented as the mean ± SD values of data from three independent experiments. *P < 0.05, **P < 0.01, ***P < 0.001, ****P < 0.0001.

RNA-sequencing (RNA-seq) was used to evaluate the gene expression in HL-60 cells after TAS4464 treatment compared to that in DMSO-treated control cells. The entire expression pattern was altered in a time-dependent manner after exposure (Fig. 4A). We selected the differentially expressed genes that were affected by TAS4464 treatment within 24 h (Fig. 4B) and predicted the upstream regulators by comparing the gene expression pattern in untreated cells to that in treated cells at 24 h (Fig. 4C). We identified candidate genes, including *TP53* and *MYC*, as key regulators by their induction in response to TAS4464. Proteomic data (Fig. 1D) also indicated similar overall alterations (Supplementary Fig. S2a) and upstream candidates as regulators (Supplementary Fig. S2b). The genetic status of *TP53* status was different in each AML cell line that we analyzed, but TAS4464 treatment demonstrated similar efficiency in all cells (Fig. 1A). The expression of apoptotic molecules, including NOXA and c-FLIP, also exhibited a similar response after TAS4464 treatment in HL-60 [p53-null], THP-1 [p53-mutant (p. Arg174fs*3)], Kasumi-1 [p53-mutant (p. Arg248Gln)] and MV-4-11 [p53-wild type] cells (Figs. 2A, 3A and Supplementary Fig. S2c). Moreover, we performed comprehensive analyses with the p53-null cell line HL-60. From the results, *MYC* was considered the most relevant upstream gene. In fact, many *MYC*-related genes were included among the differentially expressed genes in the clusters affected by TAS4464 (Fig. 4B). In addition, the actual gene expression levels after TAS4464 treatment both in the upregulated cluster (Fig. 4D) and in the downregulated cluster (Fig. 4E) were validated by qRT-PCR.

**Fig. 5:**
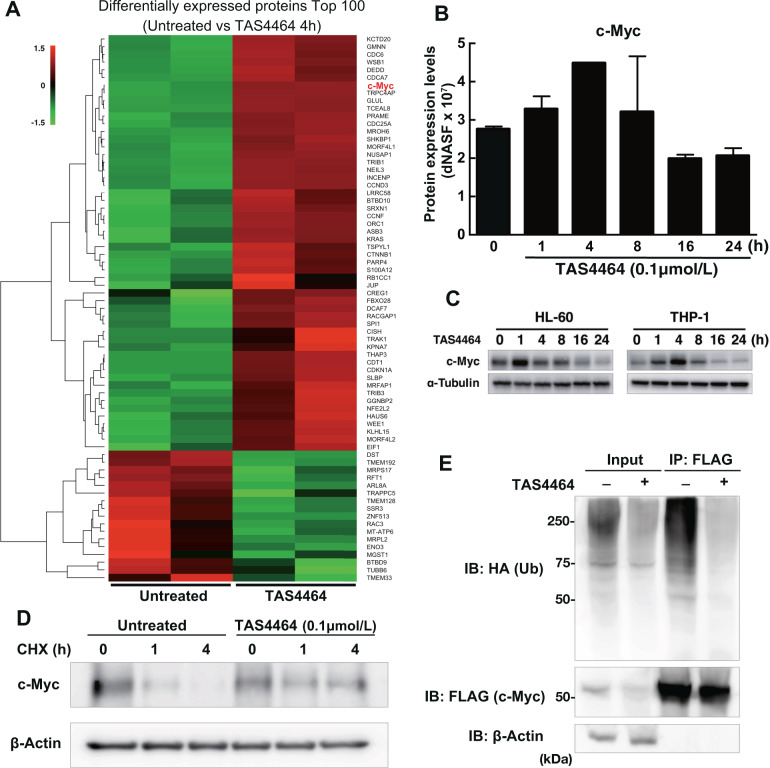
TAS4464 increases the protein stability of c-Myc. **A** The top 100 differentially expressed proteins between untreated cells and treated with 0.1 μmol L^−1^ TAS4464 for 4 h identified by non-target proteomic analysis are listed in the heatmap. **B** Changes in c-Myc protein expression levels identified in proteomic analysis during treatment with 0.1 μmol L^−1^ TAS4464 for up to 24 h. Data are presented as the mean ± SD values of data from three independent experiments. **C** Immunoblotting for c-Myc in HL-60 and THP-1 cells treated with 0.1 μmol L^−1^ TAS4464. Samples were harvested at 1, 4, 8, 16, and 24 h after treatment. **D** Cycloheximide (CHX)-chase analysis for HL-60 cells. The HL-60 cells were treated with DMSO (Untreated) or TAS4464 (0.1 μmol L^−1^) in the presence of CHX (100 μg mL^−1^) for the indicated time points (hours). Immunoblotting for c-Myc was performed to evaluate the protein stability. **E** Immunoblotting for HA-tagged ubiquitin and FLAG-tagged c-Myc after immunoprecipitation of c-Myc. 3×FLAG-c-Myc and HA-ubiquitin were transfected into HEK293T cells. After 8 h of transfection, cells were treated with MG132 (1 mM) and TAS4464 (0.1 μmol L^−1^) for the last 16 h, and the extracted proteins were subjected to ubiquitination assay for c-Myc.

Since c-Myc transcriptionally regulates downstream genes, we focused on the early time point after TAS4464 induction. While the RNA-seq data did not indicate a drastic increase in the level of *MYC* itself (Fig. 4B), our proteomic data revealed that the c-Myc protein was included among the molecules highly upregulated after 4 h of TAS4464 treatment in HL-60 cells (Fig. 5A). Throughout the time course, the c-Myc protein level obviously peaked at ~4 h after treatment (Fig. 5B). Accordingly, we verified that TAS4464 treatment resulted in transient accumulation of the c-Myc protein in both HL-60 and THP-1 cells (Fig. 5C), suggesting that TAS4464 stabilizes the c-Myc protein by facilitating its escape from the degradation machinery. Then, we confirmed the increase in c-Myc stability after TAS4464 treatment via a pulse-chase experiment (Fig. 5D). TAS4464 is a drug that suppresses the neddylation pathway; thus, we next evaluated c-Myc ubiquitination. c-Myc was precipitated with an anti-FLAG antibody (Ab) after exogenous overexpression, and ubiquitination of those proteins was assessed by immunoblotting with an anti-HA Ab on those proteins. c-Myc ubiquitination was found to be decreased after TAS4464 treatment, as we hypothesized (Fig. 5E).

### TAS4464-induced c-Myc activation transcriptionally regulates NOXA and c-FLIP level

**Fig. 6:**
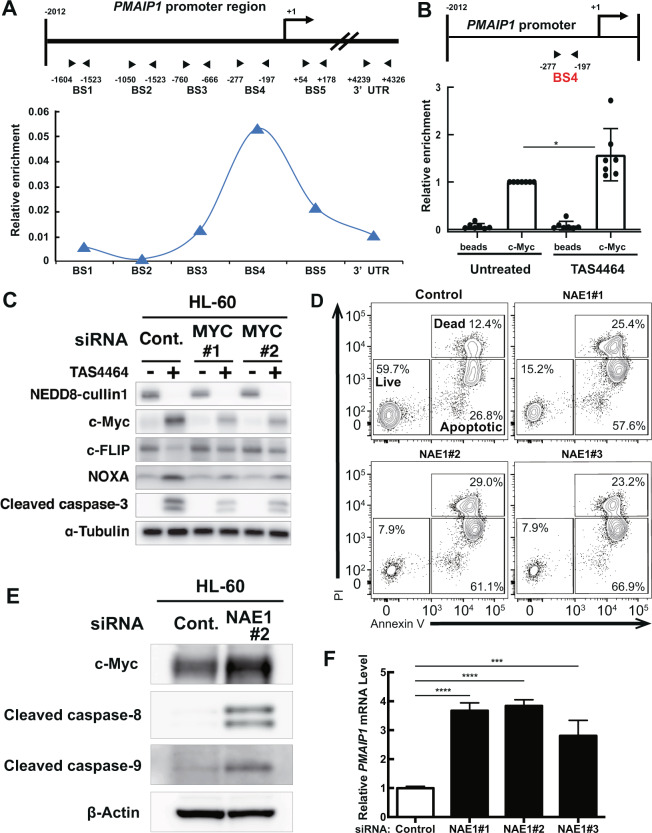
TAS4464-induced c-Myc activation transcriptionally mediates apoptotic gene expression. **A** Schematic representation of the *PMAIP1* promoter region and c-Myc enrichment at each site. The regions targeted by the primer pairs are indicated as “BS1” to “BS5” and “3′ UTR”. **B** Enrichment of c-Myc protein in the BS4 region in (**A**). The fold enrichment value compared to the untreated condition was used to evaluate the effect of TAS4464 induction. HL-60 cells were treated with TAS4464 (0.1 μmol L^−1^) for 4 h. **C** *MYC* siRNAs were transfected into HL-60 cells. After 48 h, cells were treated with 0.1 μmol L^−1^ TAS4464 for 16 h except for c-Myc detection (1 h). Total protein was extracted and then immunoblotting for NEDD8-cullin1, c-Myc, c-FLIP, NOXA and cleaved caspase-3 was performed. **D** Apoptotic cell death was evaluated by flow cytometric analysis. HL-60 cells were treated with *NAE1* siRNAs for 16 h. **E** Immunoblotting for c-Myc, Cleaved caspase-8 and Cleaved caspase-9 after *NAE1* siRNAs transfection. Cells were harvested 8 h after transfection. **F** qRT-PCR to measure the level of PMAIP*1* mRNA was performed in HL-60 cells after *NAE1* siRNA transfection at 16 h time point. Data are presented as the mean ± SD values of data from three independent experiments. *P < 0.05, ***P < 0.001, ****P < 0.0001.

c-Myc regulates *PMAIP1* and *CFLAR* expression by recruitment to their promoters even though their promoters do not contain a conventional Myc-binding consensus sequence [33, 34]. To determine whether *PMAIP1* and *CFLAR* are direct transcriptional targets of c-Myc in TAS4464-treated AML cells, we evaluated c-Myc-binding at their promoter regions by ChIP-qPCR scanning. Regarding *PMAIP1*, we found that c-Myc proteins were enriched ~200 bp upstream of the transcription start site (TSS) in HL-60 cells upon treatment with 0.1 μmol L^−1^ TAS4464 for 4 h (Fig. 6A). c-Myc binding at this location was observed even under steady-state conditions, but it was significantly increased by TAS4464 induction (Fig. 6B). In addition, we identified c-Myc binding in the *CFLAR* promoter region downstream of its TSS (Supplementary Fig. S3a). Similar to its effect on c-Myc recruitment to the *PMAIP* promoter, TAS4464 treatments tended to increase c-Myc recruitment to this region of the *CFLAR* promoter (Supplementary Fig. S3b). This TAS4464-induced recruitment was also observed in the promoter regions of the other c-Myc target molecules (Supplementary Fig. S3c); therefore, TAS4464 treatment induced global c-Myc accumulation on its transcriptional targets. To evaluate the involvement of c-Myc in the TAS4464-mediated modulation of NOXA and c-FLIP expression, we gave TAS4464 treatment to HL-60 cells with *MYC* siRNA. c-Myc knockdown diminished the both TAS4464-induced increase in NOXA and decrease in c-FLIP protein expression (Fig. 6c). Taken together, we concluded that TAS4464 treatment enhances *PMAIP1* transcription and reduces *CFLAR* transcription by recruiting c-Myc to their promoters.

Moreover, NAE1 binds to UBA3 to form the NEDD8-activating enzyme complex that neddylates target proteins. Therefore, we generated HL-60 cells with NAE1 knockdown by siRNA transfection to determine whether molecular interference with the NEDD8 pathway induces the same effects as TAS4464 treatment (Supplementary Fig. S4). Expectedly, NAE1 knockdown promoted apoptosis by activating c-Myc (Fig. 6D, E). Consequently, we confirmed caspase cleavage both in intrinsic pathway and extrinsic pathway, and downstream transcription (Fig. 6E, F).

TAS4464 has previously been reported to suppress cell growth in various malignancies [27], but its effectiveness differs across cell types. Therefore, we investigated the mechanisms by which the above-mentioned machinery contributes to solid tumor development. c-Myc accumulation resulted from TAS4464 treatment in MCF7 (breast cancer) cells, similar to the finding in AML cell lines (Supplementary Fig. S5a), and c-Myc knockout (c-Myc KO) decreased TAS4464-mediated molecular responses, such as NOXA induction, Caspase cleavage and transcriptional suppression of *CFLAR* (Supplementary Fig. S5a, b). Consistently, p53 was also activated by TAS4464 (Supplementary Fig. S5c), and both c-Myc KO and p53 knockout (p53 KO) inhibited the cell death, even though the basal survival rate was higher in control cells (Supplementary Fig. S6a, b). In particular, the spheroid culture system, which is more similar to the in vivo condition than to conventional two-dimensional cell culture conditions, exemplified the drastic suppression of TAS4464-induced cell death by single knockout targeting either c-Myc or p53 (Supplementary Fig. S6c, d). This response was partly different from that in AML cells such as p53-null HL-60 cells, therefore suggesting that the molecular effects of TAS4464 could vary depending on cell type or culture condition.

### TAS4464 exhibits antitumor activity via activation of both the intrinsic and extrinsic apoptotic pathways accompanied by increase in the NOXA and decrease in the c-FLIP level in a human AML xenograft model

**Fig. 7:**
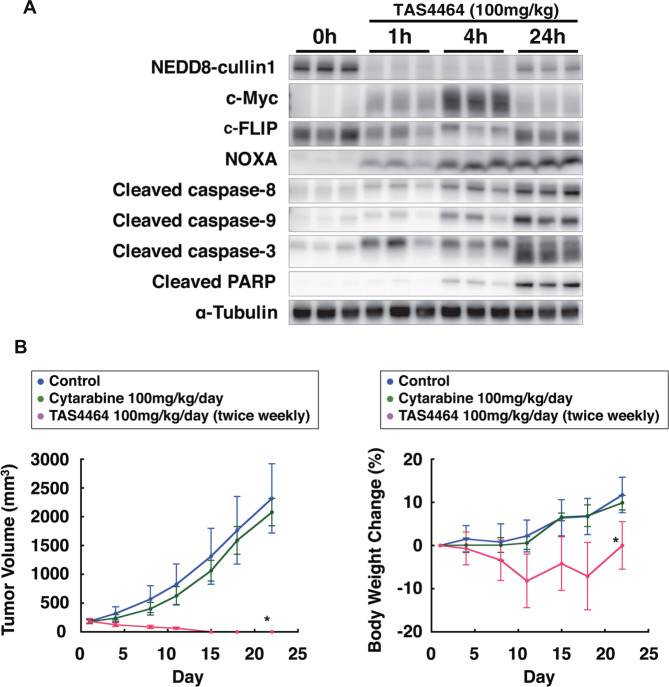
TAS4464 exerts strong antitumor activity via activation of both the intrinsic and extrinsic apoptotic pathways accompanied by an increase in the NOXA level and a decrease in the c-FLIP level in a human AML xenograft model. **A** TAS4464 (100 mg kg^−1^) was administered intravenously. Tumors were harvested at the indicated times after administration of TAS4464 and prepared for immunoblotting. **B** TAS4464 was administered intravenously twice weekly at 100 mg kg^−1^ per day. Cytarabine was administered intravenously twice a week for 3 weeks at 100 mg kg^−1^ per day. Data are presented as the mean ± SD values. *P < 0.05 in the treated group compared with the control group (Dunnett’s test).

To examine whether an increase in NOXA and decrease in c-FLIP expression occurs at concentrations therapeutically achievable in vivo, the activity of TAS4464 was evaluated in a human AML THP-1 xenograft mouse model. TAS4464 was administered intravenously (100 mg kg^−1^) to THP-1 xenograft model mice, and tumors were collected at 1, 4, and 24 h after administration of TAS4464 to assess the pharmacodynamic response. Elevation of c-Myc was observed in accordance with NAE inhibition by TAS4464, as evidenced by the changes in the amount of NEDD8-cullin binding, with the maximal effect occurring 1–4 h after administration of TAS4464 (Fig. 7A). TAS4464 treatments also resulted in the accumulation of the NOXA and a reduction in the amount of c-FLIP, and subsequently led to the activation of caspase-3, -8 and -9 (Fig. 7A). These findings further support our notion that TAS4464 induces apoptosis via both the intrinsic and extrinsic apoptotic pathways, accompanied by an increase in the NOXA and a decrease in the c-FLIP level in AML.

TAS4464 showed antitumor activity in this AML model. Intravenous administration of 100 mg kg^−1^ TAS4464 twice weekly for 3 weeks induced complete tumor remission without severe weight loss in mice, while twice-weekly administration of cytarabine, a standard chemotherapeutic agent for AML, had only a small impact on tumor growth (Fig. 7B). These results suggest that at concentrations therapeutically achievable in vivo, TAS4464 activates both the intrinsic and extrinsic apoptotic pathways accompanied by an increase in the NOXA level and a decrease in c-FLIP level, leading to antitumor activity.

## Discussion

Protein neddylation is a newly proposed posttranslational modification, and its regulation is related to various diseases including cancer [35]. The inhibition of the neddylation pathway has been proposed to suppress tumor growth in patients. In this study, we demonstrated the molecular mechanism of a novel NAE inhibitor, TAS4464, in human AML regression.

Among the proposed mechanisms of NAE inhibition in AML, we found that TAS4464 increased the NOXA level and decreased the c-FLIP level in several AML cell lines. Consistent with this observation, these molecules suppress tumor growth via apoptosis mediated through both the intrinsic and extrinsic apoptotic pathways [36, 37]. Indeed, our comprehensive analysis revealed that the Myc pathway is highly associated with the molecules affected by TAS4464 treatment. c-Myc is broadly accepted to be a protooncogene, since its activation has been observed in more than 70% of all cancers and plays roles in initiating oncogenic process and cell proliferation. The transcriptional activity of c-Myc is regulated mostly by its dimerization with Max, and dysregulation of this dimerization has been proposed as a therapeutic target for cancers [38]. On the other hand, overexpression of c-Myc leads to an apoptotic response independent of Max recruitment. For instance, c-Myc is responsible for cell death in Burkitt’s lymphoma cells deprived of autocrine factors. Epithelial cells have also been shown to be susceptible to c-Myc-mediated apoptosis. In addition, c-Myc is required for an efficient response to a variety of apoptotic stimuli, including transcription and translation inhibitors, hypoxia, heat shock, DNA damage, and cancer chemotherapeutics [39]. In cells with high c-Myc expression, p53 is activated and the metabolic status is altered [40]. Moreover, a recent study showed that NAE inhibition induces Myc accumulation followed by NOXA induction [28]. c-FLIP is another known c-Myc target molecule, and c-Myc enhances the apoptotic effect of NAE inhibition by inhibiting c-FLIP. Downregulation of c-FLIP can facilitate c-Myc-induced apoptosis. Although MLN4924 was previously reported to facilitate c-FLIP degradation through promoting its ubiquitination in head and neck cancer cells [41], the change in c-FLIP protein expression induced by TAS4464 treatment paralleled the change in the mRNA levels in AML cells. In our study, we demonstrated the involvement of c-Myc in the transcription of NOXA and c-FLIP. These pathways induce apoptosis in AML cell lines; thus, their transactivation by c-Myc could confer beneficial therapeutic effects in cancer. C-FLIP has been reported to play critical roles in AML survival, because it is highly expressed in AML cells, and its expression is correlated with poor prognosis in AML patients [4]. Thus, downregulation of c-FLIP might be a rational therapeutic approach for AML.

However, our findings regarding the antitumor activity of TAS4464 via apoptosis might not be fully explained by c-Myc regulation alone. First, both cultured tumor cells and xenograft tumor cells exhibited c-Myc activation, but the duration was limited to the initial few hours. However, the effect of TAS4464 treatment presumably lasted days in vitro and weeks in vivo. Second, c-Myc inhibition did not completely reverse the altered NOXA and c-FLIP expression levels to the basal levels, and TAS4464 retained its antitumor activity under c-Myc inhibition. Third, p53 knockdown also showed the inhibitory effect on TAS4464-mediated apoptosis similar to that of c-Myc knockdown in certain cell types. Therefore, our results indicate that c-Myc regulation could act cooperatively with the other possible regulators, such as mechanisms involving the *TP53* pathway or *E2F* family. Further investigation is needed to clarify their mutual coordination upon a TAS4464-mediated apoptosis.

In conclusion, we demonstrated TAS4464-mediated activation of apoptotic pathways in AML cells. This regulation was mediated by NOXA and c-FLIP, resulting in activation of caspases in both the intrinsic and extrinsic pathways. Our study provides crucial evidence supporting TAS4464 as an antitumor agent.

## Materials and methods

### Chemical compounds and antibodies

TAS4464 was designed and synthetized at Taiho Pharmaceutical Co., Ltd. and cytarabine (cytosine beta-D-arabinofuranoside hydrochloride) and the anti-Actin antibody (#A2066) were purchased from Sigma-Aldrich Co., LLC. Anti-cleaved caspase-3 (#9664), anti-cleaved caspase-6 (#9761), anti-cleaved caspase-7 (#8438), anti-cleaved caspase-8 (#9496), anti-cleaved caspase-9 (#9501, #20750), anti-cleaved PARP (#9541), anti-Bak (#3814), anti-Bax (#2772), anti-Mcl-1 (#4572), anti-Puma (#4976), anti-Bim (#2819), anti-Bcl-2 (#2872), anti-Bcl-xl (#2762), anti-Bcl2l10 (#3869), anti-XIAP (#2042), and anti-c-Myc (#5605) antibodies were purchased from Cell Signaling Technology, Inc. Anti-c-FLIP (sc-5276), anti-FAS (sc-715), anti-FAS-L (sc-957) and anti-c-Myc (sc-40) antibodies were purchased from Santa Cruz Biotechnology, Inc. Anti-NEDD8 (ab81264), anti-NOXA (ab13654), anti-c-Myc (ab32072), and anti-α-Tubulin (ab4047) antibodies were purchased from Abcam plc.

### Cell-cycle analysis and apoptosis analysis

Cells were treated with TAS4464 for the indicated times. For cell cycle analysis, the cells were harvested and stained with a BD Cycletest™ Plus DNA Reagent Kit (BD Biosciences). Cell-cycle distributions were determined by using a flow cytometer [FACSVerse^TM^, (BD Biosciences)]. For apoptosis analysis, the harvested cells were stained a FITC Annexin V Apoptosis Detection Kit I (BD Pharmingen ^TM^) for HL-60 cells or with an Annexin V -633 Apoptosis Detection Kit^TM^ (nacalai tesque^TM^) for MCF7 cells according to the manufacturer’s protocol. In brief, 1 × 10^5^ cells were incubated with 5 μl of Annexin V and 5 μl of propidium iodide at room temperature in the dark. The cells were analyzed immediately by flow cytometry [FACSCanto^TM^II, (BD Biosciences)] immediately.

### Chromatin immunoprecipitation (ChIP)

HL-60 cells (1 × 10^6^) were seeded and were fixed 2 days later with 0.5% formaldehyde in IMDM for 10 min at 37 °C. The reaction was quenched by the addition of 700 μl of 2.5 M glycine, and the cells were washed with PBS. Pelleted nuclei were dissolved in lysis buffer [50 mM Tris-HCl (pH 8.0), 1% SDS and 10 mM EDTA] and sonicated in a UD-201ultrasonic disruptor (Tomy) with 50 cycles of sonication for 5 s followed by rest for 5 s at the power specified in programmed memory setting 4. Ten micrograms of an anti-c-Myc antibody mixture [a mixture of anti-c-Myc (SC-40) and anti-c-Myc (ab32072)] was separately prebound to Dynabeads Protein A/G (Invitrogen) and then added individually to the diluted chromatin complexes in parallel aliquots. The samples were incubated overnight at 4 °C, washed, and eluted for 6 h at 65 °C in ChIP elution buffer [40 m M Tris-HCl (pH 6.5), 0.1 M NaHCO_3_, 1% SDS, 0.2 M NaCl, 10 mM EDTA and 10 μg μL^−1^ proteinase K]. Precipitated chromatin fragments were cleaned using a PCR purification kit (Qiagen). The samples were analyzed by qPCR at the region sites in the *PMAIP1* and *CFLAR* promoters. The primer sequences used are listed in Supplemental Table 1.

### RNA-sequence analysis and non-target proteomics analysis

HL-60 cells were either not treated (control) or treated with TAS4464 (0.1 μmol L^−1^) for 24 or 48 h. Then, RNA was isolated from the cell samples using a phenol-guanidinium isothiocyanate reagent according to the manufacturer’s protocol [42]. RNA was precipitated from the aqueous phase with isopropanol, and proteins were precipitated from the phenol/ethanol phase by the addition of acetone. A total of single-end-read RNA-seq tags were generated using a HiSeq 2000 sequencer according to the standard protocol. The generated sequence tags were mapped onto the human genome sequence (hg19 from the University of California Santa Cruz Genome Browser) using the Eland program (Illumina). RNA-seq data are available in Dryad database (doi:10.5061/dryad.hqbzkh1f6) [43]. The protein extract was digested, and the peptides were analyzed by LC-MS/MS (Q Exactive Orbitrap HF-X). Differential expression was estimated and analyzed with the TCC software package in R/Bioconductor 3.1 for each library with reference to the control. The mass spectrometry proteomics data have been deposited in the ProteomeXchange Consortium (http://proteomecentral.proteomexchange.org) via the jPOST partner repository (http://jpostdb.org) with the dataset identifier PXD021012/ JPST000944.

### In vivo efficacy studies

THP-1 cells were subcutaneously implanted into 5-week-old male BALB/c nude mice (CLEA Japan, Inc.) and allowed to grow. Six animals were assigned to each group. TAS4464 formulated in 5% (w/v) glucose solution (Otsuka Pharmaceutical Factory, Inc.) was administered intravenously twice weekly for three weeks. Cytarabine formulated in normal saline solution (Otsuka Pharmaceutical Factory, Inc.) was administered intravenously twice weekly at a dosage of 100 mg kg^−1^ per day for three weeks. The dose of cytarabine was equivalent to the maximum tolerated dose determined by internal evaluation. The tumor volume (TV) was calculated with the formula [length × (width)^2^]/2. During the treatment period, the TV and weight of the mice were measured twice weekly. Dunnett’s test was used as to assess the differences in TVs between the drug-treated groups and the control group. *P* < 0.05 was considered statistically significant. For pharmacodynamic analysis, tumors were harvested at the indicated time points after the administration of TAS4464. The excised tumors were homogenized in lysing matrix D (MP Biomedicals, LLC) containing lysis buffer, and lysates were prepared for immunoblot analysis. All animal experiments were performed with the approval of the institutional animal care and use committee of Taiho Pharmaceutical Co., Ltd. and carried out according to the Taiho Pharmaceutical Co., Ltd. guidelines for animal experiments.

The following detailed methods are listed in Supplementary Materials and methods, including the Cell lines and cell cultures, cell viability assay and quantification of apoptosis induction, Immunoblot analysis, RNA interference, qRT-PCR, Spheroid culture, cycloheximide (CHX)-chase analysis, Ubiquitination assay for c-Myc, Generation of knockout cell lines, and Statistical analysis.

## Supplementary information

Supplemental Figure and Legend

Supplemental Table 1

Supplemental Material and Methods

## References

[1] Kumar CC (2011). Genetic abnormalities and challenges in the treatment of acute myeloid leukemia. Genes Cancer.

[2] Sweet K, Lancet JE (2014). Novel therapeutics in acute myeloid leukemia. Curr Hematol Malig Rep.

[3] Konopleva M, Zhao S, Hu W, Jiang S, Snell V, Weidner D (2002). The anti-apoptotic genes Bcl-X(L) and Bcl-2 are over-expressed and contribute to chemoresistance of non-proliferating leukaemic CD34^+^ cells. Br J Haematol.

[4] McLornan D, Hay J, McLaughlin K, Holohan C, Burnett AK, Hills RK (2013). Prognostic and therapeutic relevance of c-FLIP in acute myeloid leukaemia. Br J Haematol.

[5] Mehta SV, Shukla SN, Vora HH (2013). Overexpression of Bcl2 protein predicts chemoresistance in acute myeloid leukemia: its correlation with FLT3. Neoplasma..

[6] Li XX, Zhou JD, Wen XM, Zhang TJ, Wu DH, Deng ZQ (2019). Increased MCL-1 expression predicts poor prognosis and disease recurrence in acute myeloid leukemia. Onco Targets Ther.

[7] DiNardo CD, Pratz K, Pullarkat V, Jonas BA, Arellano M, Becker PS (2019). Venetoclax combined with decitabine or azacitidine in treatment-naive, elderly patients with acute myeloid leukemia. Blood..

[8] Gong L, Yeh ET (1999). Identification of the activating and conjugating enzymes of the NEDD8 conjugation pathway. J Biol Chem.

[9] Petroski MD, Deshaies RJ (2005). Function and regulation of cullin-RING ubiquitin ligases. Nat Rev Mol Cell Biol.

[10] Soucy TA, Smith PG, Rolfe M (2009). Targeting NEDD8-activated cullin-RING ligases for the treatment of cancer. Clin Cancer Res.

[11] Pan Y, Xu H, Liu R, Jia L (2012). Induction of cell senescence by targeting to Cullin-RING Ligases (CRLs) for effective cancer therapy. Int J Biochem Mol Biol.

[12] Read MA, Brownell JE, Gladysheva TB, Hottelet M, Parent LA, Coggins MB (2000). Nedd8 modification of cul-1 activates SCF(beta(TrCP))-dependent ubiquitination of IkappaBalpha. Mol Cell Biol.

[13] Barkett M, Gilmore TD (1999). Control of apoptosis by Rel/NF-kappaB transcription factors. Oncogene..

[14] Kamitani T, Kito K, Nguyen HP, Yeh ET (1997). Characterization of NEDD8, a developmentally down-regulated ubiquitin-like protein. J Biol Chem.

[15] Enchev RI, Schulman BA, Peter M (2015). Protein neddylation: beyond cullin-RING ligases. Nat Rev Mol Cell Biol.

[16] Hori T, Osaka F, Chiba T, Miyamoto C, Okabayashi K, Shimbara N (1999). Covalent modification of all members of human cullin family proteins by NEDD8. Oncogene..

[17] Pan ZQ, Kentsis A, Dias DC, Yamoah K, Wu K (2004). Nedd8 on cullin: building an expressway to protein destruction. Oncogene..

[18] Soucy TA, Dick LR, Smith PG, Milhollen MA, Brownell JE (2010). The NEDD8 conjugation pathway and its relevance in cancer biology and therapy. Genes Cancer.

[19] Xie P, Zhang M, He S, Lu K, Chen Y, Xing G (2014). The covalent modifier Nedd8 is critical for the activation of Smurf1 ubiquitin ligase in tumorigenesis. Nat Commun.

[20] Li L, Wang M, Yu G, Chen P, Li H, Wei D, et al. Overactivated neddylation pathway as a therapeutic target in lung cancer. J Natl Cancer Inst. 2014;106:dju083. 10.1093/jnci/dju083 24853380

[21] Xie P, Yang JP, Cao Y, Peng LX, Zheng LS, Sun R (2017). Promoting tumorigenesis in nasopharyngeal carcinoma, NEDD8 serves as a potential theranostic target. Cell Death Dis.

[22] Swords RT, Erba HP, DeAngelo DJ, Bixby DL, Altman JK, Maris M (2015). Pevonedistat (MLN4924), a First-in-Class NEDD8-activating enzyme inhibitor, in patients with acute myeloid leukaemia and myelodysplastic syndromes: a phase 1 study. Br J Haematol.

[23] Shah JJ, Jakubowiak AJ, O’Connor OA, Orlowski RZ, Harvey RD, Smith MR (2016). Phase I study of the novel investigational NEDD8-activating enzyme inhibitor pevonedistat (MLN4924) in patients with relapsed/refractory multiple myeloma or lymphoma. Clin Cancer Res.

[24] Sarantopoulos J, Shapiro GI, Cohen RB, Clark JW, Kauh JS, Weiss GJ (2016). Phase I study of the investigational NEDD8-activating enzyme inhibitor pevonedistat (TAK-924/MLN4924) in patients with advanced solid tumors. Clin Cancer Res.

[25] Bhatia S, Pavlick AC, Boasberg P, Thompson JA, Mulligan G, Pickard MD (2016). A phase I study of the investigational NEDD8-activating enzyme inhibitor pevonedistat (TAK-924/MLN4924) in patients with metastatic melanoma. Investig New Drugs.

[26] Lockhart AC, Bauer TM, Aggarwal C, Lee CB, Harvey RD, Cohen RB (2019). Phase Ib study of pevonedistat, a NEDD8-activating enzyme inhibitor, in combination with docetaxel, carboplatin and paclitaxel, or gemcitabine, in patients with advanced solid tumors. Investig New Drugs.

[27] Yoshimura C, Muraoka H, Ochiiwa H, Tsuji S, Hashimoto A, Kazuno H (2019). TAS4464, a highly potent and selective inhibitor of NEDD8-activating enzyme, suppresses neddylation and shows antitumor activity in diverse cancer models. Mol Cancer Ther.

[28] Knorr KL, Schneider PA, Meng XW, Dai H, Smith BD, Hess AD (2015). MLN4924 induces Noxa upregulation in acute myelogenous leukemia and synergizes with Bcl-2 inhibitors. Cell Death Differ.

[29] Kiechle FL, Zhang X (2002). Apoptosis: biochemical aspects and clinical implications. Clin Chim Acta.

[30] Stennicke HR, Salvesen GS (1998). Properties of the caspases. Biochim Biophys Acta.

[31] Muraoka H, Yoshimura C, Kawabata R, Tsuji S, Hashimoto A, Ochiiwa H (2019). Activity of TAS4464, a novel NEDD8 activating enzyme E1 inhibitor, against multiple myeloma via inactivation of nuclear factor kappaB pathways. Cancer Sci.

[32] Krueger A, Baumann S, Krammer PH, Kirchhoff S (2001). FLICE-inhibitory proteins: regulators of death receptor-mediated apoptosis. Mol Cell Biol.

[33] Wirth M, Stojanovic N, Christian J, Paul MC, Stauber RH, Schmid RM (2014). MYC and EGR1 synergize to trigger tumor cell death by controlling NOXA and BIM transcription upon treatment with the proteasome inhibitor bortezomib. Nucleic Acids Res.

[34] Ricci MS, Jin Z, Dews M, Yu D, Thomas-Tikhonenko A, Dicker DT (2004). Direct repression of FLIP expression by c-myc is a major determinant of TRAIL sensitivity. Mol Cell Biol.

[35] Zhou L, Jiang Y, Luo Q, Li L, Jia L (2019). Neddylation: a novel modulator of the tumor microenvironment. Mol Cancer.

[36] Bogenberger J, Whatcott C, Hansen N, Delman D, Shi CX, Kim W (2017). Combined venetoclax and alvocidib in acute myeloid leukemia. Oncotarget..

[37] Conti S, Petrungaro S, Marini ES, Masciarelli S, Tomaipitinca L, Filippini A (2016). A novel role of c-FLIP protein in regulation of ER stress response. Cell Signal.

[38] Chen H, Liu H, Qing G (2018). Targeting oncogenic Myc as a strategy for cancer treatment. Signal Transduct Target Ther.

[39] Hoffman B, Liebermann DA (2008). Apoptotic signaling by c-MYC. Oncogene..

[40] Phesse TJ, Myant KB, Cole AM, Ridgway RA, Pearson H, Muncan V (2014). Endogenous c-Myc is essential for p53-induced apoptosis in response to DNA damage in vivo. Cell Death Differ.

[41] Zhao L, Yue P, Lonial S, Khuri FR, Sun SY (2011). The NEDD8-activating enzyme inhibitor, MLN4924, cooperates with TRAIL to augment apoptosis through facilitating c-FLIP degradation in head and neck cancer cells. Mol Cancer Ther.

[42] Kawashima Y, Miyata J, Watanabe T, Shioya J, Arita M, Ohara O (2019). Proteogenomic analyses of cellular lysates using a phenol-guanidinium thiocyanate reagent. J Proteome Res.

[43] Tanaka T. RNA-seq data from TAS4464, a NEDD8-activating enzyme inhibitor, activates both intrinsic and extrinsic apoptotic pathways via c-Myc-mediated regulation in acute myeloid leukemia. Dryad Digital Repository 2020. Deposited 9 December 2020. 10.5061/dryad.hqbzkh1f6. 10.1038/s41388-020-01586-4 PMC7892340 33420360

