# Supplementary material for: TAS4464, a NEDD8-activating enzyme inhibitor, activates both intrinsic and extrinsic apoptotic pathways via c-Myc-mediated regulation in acute myeloid leukemia
Source: Oncogene. 2021 Jan 8;40(7):1217–30. doi: 10.1038/s41388-020-01586-4 (PMC7892340; doi:10.1038/s41388-020-01586-4)
Supplement: Supplementary file 1 — Supplemental Figure and Legend [file 41388_2020_1586_MOESM1_ESM.docx]

**Supplementary Figure S1**. **Cell cycle analysis after TAS4464 treatment.**

(a) Cell cycle profiles were evaluated by flow cytometric analysis. HL-60 and THP-1 cells were treated with 0.1 μmol L^-1^ TAS4464 for 0, 8, 16 and 24 hours. (b) Total percentages of cells in each cell cycle phase shown in Fig. S1a.

**Supplementary Figure S2**. **Comprehensive analysis based on proteomics data in HL-60 cells after TAS4464 treatment and the apoptotic pathway response in MV-4-11 and Kasumi-1 cells.**

(a) Principal component analysis of proteomics data shows the clusters of HL-60 cells depending on the duration of TAS4464 treatment. DMSO (control) was used for the untreated condition. (b) Based on the differences in protein expression between untreated cells and cells treated with TAS4464 for 24 hours, upstream regulators were predicted using Ingenuity Pathway Analysis. (c) MV-4-11 and Kasumi-1 cells were treated with 0.1 μmol L^-1^ TAS4464 for 1, 4, 8, 16 and 24 hours, and total protein was extracted. Changes in protein levels were detected with the indicated antibodies.

**Supplementary Figure S3**. **c-Myc binding in the promoter regions of CFLAIR and the other target genes.**

(a) Schematic representation of the *CFLAIR* promoter region and c-Myc enrichment at each site. The regions targeted by the primer pairs are indicated as “BS1” to “BS7” and “3’ UTR”. (b) The relative enrichment value compared to the untreated condition was used to evaluate the effect of TAS4464 induction at the site downstream of the TSS. HL-60 cells were treated with TAS4464 (0.1 μmol L^-1^) for 4 hours. (c) The relative enrichment value compared to the untreated condition was used to assess c-Myc binding at the promoter regions of *CDKN1A, E2F1, E2F2* and *IDH2* after treatment of TAS4464 (0.1 μmol L^-1^) for 4 hours. Data are presented as the mean ± SD values of data from three independent experiments. * *P* < 0.05

**Supplementary Figure S4**. **Treatment with siRNA targeting *NAE1.***

*NAE1* siRNAs were transfected into HL-60 cells. Cells were harvested after 16 hours, and *NAE1* levels were evaluated by qRT-PCR. Data are presented as the mean ± SD values of data from three independent experiments. ***P* < 0.01, ****P* < 0.001.

**Supplementary Figure S5**. **c-Myc and p53 knockout by CRISPR-Cas9 genome editing in MCF7 cells.**

(a) c-Myc was knocked out by the CRISPR-Cas9 system in MCF7 cells. Immunoblotting for c-Myc, NOXA, Cleaved caspase-8 and Cleaved caspase-9 was performed in unedited or c-Myc KO MCF7 cells treated with or without TAS4464 (0.1 μmol L^-1^) for 36 hours. (b) qRT-PCR was performed to measure the level of *CFLAR* mRNA was in unedited or c-Myc KO MCF7 cells treated with or without TAS4464 (0.1 μmol L^-1^) for 8 hours. Data are presented as the mean ± SD values of data from three independent experiments. ***P* < 0.01. (c) p53 was knocked out by CRISPR-Cas9 system in MCF7 cells. Immunoblotting for p53 was performed in cells treated with or without TAS4464 (0.1 μmol L^-1^) for 8 hours.

**Supplementary Figure S6**. **Contribution of c-Myc and p53 to TAS4464-induced cell death.**

(a) Apoptotic cell death was evaluated in c-Myc KO and p53 KO cells by flow cytometric analysis. MCF7 cells were treated with TAS4464 (0.1 μmol L^-1^) for 36 hours. (b) Total percentages of cells with each status shown in Fig. S6a. (c) Representative images of 3D-cultured MCF7 tumor spheroids. Bright field images and PI and Hoechst staining images of each cell line treated with or without TAS4464 (0.1 μmol L^-1^) for 24 hours. (d) Fluorometric quantification of cell death in tumor spheroids. Data are presented as the mean ± SD values of data from three independent experiments. ***P* < 0.01, ****P* < 0.001, *****P* < 0.0001.
