# Supplementary material for: TAS4464, a NEDD8-activating enzyme inhibitor, activates both intrinsic and extrinsic apoptotic pathways via c-Myc-mediated regulation in acute myeloid leukemia
Source: Oncogene. 2021 Jan 8;40(7):1217–30. doi: 10.1038/s41388-020-01586-4 (PMC7892340; doi:10.1038/s41388-020-01586-4)
Supplement: Supplementary file 2 — Supplemental Table 1 [file 41388_2020_1586_MOESM2_ESM.docx]

**Supplementary Table 1 ChIP primer sequences**

The listed primers were used for ChIP-qPCR to detect c-Myc binding.

|  | **Gene name** | **Sequence** |
| --- | --- | --- |
|  | PMAIP1_BS1_Forward | CTTCGACCACACTTCAAACCT |
|  | PMAIP1_BS1_Reverse | GCATCTCTTGGAAAACCAGAA |
|  | PMAIP1_BS2_Forward | TTCGGCGAAAACACACATT |
|  | PMAIP1_BS2_Reverse | GAAAATTGCCCAAGTTCACTT |
|  | PMAIP1_BS3_Forward | CTCGCCAAACATTATGCAAA |
|  | PMAIP1_BS3_Reverse | CGAGTGGATCGTTATCATATGG |
|  | PMAIP1_BS4_Forward | GACGACGTCCAGCGTTTG |
|  | PMAIP1_BS4_Reverse | GCCCCGAAATTACTTCCTTAC |
|  | PMAIP1_BS5_Forward | CACCGTGTGTAGTTGGCATC |
|  | PMAIP1_BS5_Reverse | AACCTCAGCCTCCAACTGG |
|  | PMAIP1_3'UTR_Forward | AGGCAGCTATTTTACCATCTGG |
|  | PMAIP1_3'UTR_Reverse | GTTTACTGCCACAGTATCAACTTTT |
|  | CFLAR_BS1_Forward | TCTCCTCTGGTGTCTCAGCA |
|  | CFLAR_BS1_Reverse | CCAGACTGCTGCAACTCAGA |
|  | CFLAR_BS2_Forward | AAGGATCACTTGAGGCCAGC |
|  | CFLAR_BS2_Reverse | TTCCCGCTCTCAAACCATCC |
|  | CFLAR_BS3_Forward | TCGCTTGAACTACCAAGGCG |
|  | CFLAR_BS3_Reverse | GGGTGGATGTTCGACACAAGT |
|  | CFLAR_BS4_Forward | ACTTGTGTCGACATCCACCC |
|  | CFLAR_BS4_Reverse | CCGTGGTCCTTGTTGTTCTA |
|  | CFLAR_BS5-Forward | ACTTGTGTCGACATCCACCC |
|  | CFLAR_BS5_Reverse | CCGTGGTCCTTGTTGTCTCA |
|  | CFLAR_BS6_Forward | TTACCACCCAGAGACACGC |
|  | CFLAR_BS6_Reverse | AGAACCTCTGCCTGCTGAAC |
|  | CFLAR_BS7_Forward | TGAGAGACGGATGGTAGGCA |
|  | CFLAR_BS7_Reverse | CTGCTCAACTCCAGCTGACA |
|  | CFLAR_3'UTR_Forward | GGGCAATGAAGTGAGAGCCA |
|  | CFLAR_3'UTR_Reverse | GGGTCTTGCTCTGTCACACA |
|  | CFLAR_BS_159_Forward | GTGTAGGAGAGAAGCGCCGCGAAC |
|  | CFLAR_BS_300_Reverse | GGACTCTCCTGCCGCTGCCACCTC |
|  | p21 -194/+88 _Forward | ACCGGCTGGCCTGCTGGAACT |
|  | p21 -194/+88_Reverse | TCTGCCGCCGCTCTCTCACCT |
|  | IDH2_Forward | GCCGGCTAGGACTTGGTGAGG |
|  | IDH2_Reverse | CCTCGGCCTCGCAAAGCACT |
|  | E2F1_Forward | AGGAACCGCCGCCGTTGTTCCCG |
|  | E2F1_Reverse | CTGCCTGCAAAGTCCCGGCCACTT |
|  | E2F2_Forward | GGCGGATCATGAGGTCAGGAGA |
|  | E2F2_Reverse | CCTCAACTGATCCACCCACCTC |
