# Supplementary material for: TAS4464, a NEDD8-activating enzyme inhibitor, activates both intrinsic and extrinsic apoptotic pathways via c-Myc-mediated regulation in acute myeloid leukemia
Source: Oncogene. 2021 Jan 8;40(7):1217–30. doi: 10.1038/s41388-020-01586-4 (PMC7892340; doi:10.1038/s41388-020-01586-4)
Supplement: Supplementary file 3 — Supplemental Material and Methods [file 41388_2020_1586_MOESM3_ESM.docx]

**Supplementary Materials and Methods.**

**Cell lines and cell cultures**

ML-2, OCI-AML2, SKM-1 and SHI-1 cells were obtained from the German Collection of Microorganisms and Cell Cultures (DMSZ). NOMO-1, P31/FUJ and PL-21 cells were obtained from the Health Science Research Resources Bank (HSRRB). Kasumi-1, GDM-1, HL-60, KG-1, MV4-11 and THP-1 cells were obtained from the American Type Culture Collection (ATCC). KG-1a cells were obtained from the European Collection of Authenticated Cell Cultures (ECCAC). All cell lines were cultured in the recommended media and with the recommended serum concentrations, according to the instructions accompanying the cell lines. All cell lines were reauthenticated via short tandem repeat–based DNA profiling.

**Cell viability assay and quantification of apoptosis induction**

Cancer cells were plated in a 96-well plate and incubated overnight. For the cell viability assay, TAS4464 was added, and cells were incubated for 3 days. Cellular proliferation was evaluated using CellTiter-Glo (Promega Corp.). For quantification of apoptosis induction, cells were pretreated with 1 μmol L^-1^ z-IETD-FMK (AMS Biotechnology Ltd.) alone, 1 μmol L^-1^ z-LEHD-FMK (AMS Biotechnology Ltd.) alone, or a combination of these inhibitors for 1 hour. Then, TAS4464 was added, and cells were incubated for 16 hours. Apoptosis induction was evaluated using Caspase 3/7 Glo (Promega Corp.). These signals were normalized by the cell viability values determined by MultiTox-Fluor Reagent (Promega Corp.)

**Immunoblot analysis**

Cells were lysed in lysis buffer supplemented with protease inhibitor cocktail and phosphatase inhibitor cocktail. Proteins were separated by SDS-PAGE and transferred to polyvinylidene fluoride membranes. Membranes were sequentially blocked with blocking reagent, reacted with the appropriate primary antibodies, and then incubated with horseradish peroxidase–conjugated secondary antibodies (Cell Signaling Technology, Inc., Agilent Technologies, Inc. and GE Healthcare Inc.). Immunoreactive bands were visualized by luminol-based enhanced chemiluminescence (Thermo Fisher Scientific Inc.). Luminescence images were acquired with a LAS-4000 Mini imaging system (Fuji Photo Film Co., Ltd.) or an Amersham Imager 600 system (GE Healthcare Inc.)

**RNA interference**

siRNAs oligonucleotides against MYC (#1: s9130, #2: s9131) and a nontargeting control siRNA oligonucleotide (cat. no. 4390884) were purchased from Ambion. NAE1 (HSS113131, HSS113132, HSS113133) was purchased from Invitrogen. siRNAs were transfected into HL-60 cells with NucleofectorTM I (Amaxa Biosystems) in accordance with protocol T-019 using Cell Line NucleofectorTM Kit V (Amaxa Biosystems). At 48 hours after electroporation, TAS4464 was added, and cells were harvested at the indicated time points and subjected to immunoblot analysis.

**Quantitative reverse-transcribed polymerase chain reaction (qRT-PCR)**

The c-FLIP primer (ID: Hs00153439_m1), NOXA primer (ID: Hs00560402_m1) and 18S rRNA primer (ID: Hs99999901_s1) sequences were purchased from Thermo Fisher Scientific Inc. Cells were treated with TAS4464 for the indicated times. After the cells were harvested, total RNA was isolated with a RNeasy Plus Mini Kit (Qiagen) and were subsequently reverse transcribed into cDNA with SuperScript^TM^ III RT (Thermo Fisher Scientific Inc.). Mixtures for qRT-PCR were prepared with TaqMan^®^ Gene Expression Assays (Thermo Fisher Scientific Inc.) following the manufacturer’s protocol. qRT-PCR was performed in a QuantStudio^TM^ 7 Flex System (Thermo Fisher Scientific Inc.) with the default Fast mode setting (stage 1; 95.0 °C for 20 sec, stage 2; 40 cycles at 95.0 °C for 1 sec and 60.0 °C for 20 sec).

**Spheroid culture**

The MCF7 wild-type, c-Myc KO and p53 KO cells generated by CRISPR-Cas9 genome editing were initially cultured on 10cm dishes in DMEM supplemented with 10% FBS as well as penicillin and streptomycin. A total of 10,000 cells were embedded in 40 µl of Matrigel and spotted in a well of a 24-well plate. The cells were cultured in MammoCult Human Medium (Stem Cell Technologies, 05620) for 4 days until spheroids were formed, and then TAS4464 was added to the culture for 1 day. Spheroids were stained with PI (10 ug ml^-1^) and Hoechst (5 ug ml^-1^) in PBS for 30 minutes at 37°C without fixation. Images of the spheroids were acquired with a KEYENCE BZ-X710 microscope after three washes with PBS. The areas of spheroids stained with PI or Hoechst was determined from digitized images in BZ-X Analyzer (KEYENCE). The apoptotic effect of TAS4464 was evaluated by calculating the ratio of area of PI-positive spheroids to that of Hoechst-positive spheroids. All ratios calculated for different conditions were normalized with respect to the ratio in MCF7 wild-type cells without TAS4464 treatment.

**Cycloheximide (CHX)-chase analysis**

To assess the protein stability of c-Myc, CHX-chase analysis was performed. The HL-60 cells were incubated with or without TAS4464 (0.1 μmol L^-1^) in the presence of CHX (100 μg ml^-1^) to block protein synthesis. The cells were then harvested at 0, 1, and 4 hours post-CHX treatment. Total protein (6 μg of whole-cell extract) was separated by SDS-PAGE and subjected to the immunoblot analysis with anti-c-Myc and anti-β-actin antibodies.

**Ubiquitination assay for c-Myc**

Immunoprecipitation of poly-ubiquitinated proteins was performed as described previously [1, 2], with some modifications. HEK293T cells were transfected with plasmids encoding 3xFLAG-c-Myc and HA-ubiquitin using Lipofectamine 2000 reagents (ThermoFisher). To generate 3x FLAG-c-Myc vector, c-Myc coding sequence from pMVs-hc-Myc (addgene) was directly fused to the end of 3xFLAG peptide sequence of p3xFLAG-CMV10 (Sigma-Aldrich, St Louis, MO, USA) and cloned using NEbuilder HiFi DNA assembly cloning kit (New England BioLabs, Ipswich, MA, USA). HA-ubiquitin plasmid construct was kindly provided by prof. Carol Prives at Columbia University. Eight hours after transfection, cells were treated with MG132 (1 mM) and TAS4464 (0.1μmol L^-1^) for 15 h, then lysed in lysis buffer [50 mM Tris·HCl (pH 7.5), 150 mM NaCl, 1% Triton X-100, 0.1% SDS, 0.5% sodium deoxycholate, 10% glycerol and protease inhibitors]. Whole-cell extracts were precleared with Protein G sepharose beads (Cytiva) for 1 hour and then extracts were subjected to immunoprecipitation with anti-FLAG antibody-conjugated beads (Sigma-Aldrich Co., LLC) for 1 hour. After stringently washing, the immunoprecipitates were eluted with SDS sample buffer. All of the immune complexes were resolved by SDS-PAGE, followed by immunoblot analysis.

**Generation of knockout cell lines**

The protocol used for the CRISPR/Cas9 system was consistent with that reported by Cong et al [3]. The backbone vector pX458 pSpCas9(BB)-2A-GFP was obtained from Addgene. The target guide RNA sequences were designed at the MYC (5’-CTATGACCTCGACTACGACT-3’) and p53 genome (5’-CGTCGAGCCCCCTCTGAGTC-3’). These oligos were annealed and　phosphorylated using T4 DNA Ligase Reaction Buffer and T4 Polynucleotide Kinase (New England Biolabs) at 37℃ for 30 min and 95℃ for 5 min. pX458 was digested using BbsI (Thermo Fisher Scientific Inc.) at 37℃ for 30 min and purified using a QIAquick Gel Extraction Kit (QIAGEN). The ligation reaction of pX458 and the annealed oligos was performed at room temperature for 10 min using a Quick Ligation Kit (New England
Biolabs). Then, the ligated reactants were purified using PlasmidSafe exonuclease (Cambio) at 37℃ for 30 min. The plasmids were transformed into Stbl3, and amplified plasmids were purified using NucleoBond Xtra Midi (Takara). Each plasmid was transfected into MCF7 using Lipofectamine 2000 (Thermo Fisher Scientific Inc.) according to the manufacturer’s protocol. The plasmid-expressing MCF7 were selected by GFP soating using BD FACS Aria Ⅲ ether to establish the monoclonal cell lines.

**Statistical analysis**

Statistical significance of the differences in numerical data was assessed with a Student’s t-test and ANOVA with Tukey’s test or Dunnett's test (Xenograft model). All tests were two-tailed and a P-value below 0.05 was considered significant. All data analyses were performed using Microsoft Excel version 14.6.2 (Microsoft Corporation, Redmond, Washington), GraphPad Prism version 8.4.3 and R software ([www.r-project.org/](http://www.r-project.org/)).

**Reference**

1 Matsuzaki H, Daitoku H, Hatta M, Tanaka K, Fukamizu A. Insulin-induced phosphorylation of FKHR (Foxo1) targets to proteasomal degradation. Proc Natl Acad Sci U S A 2003; 100: 11285-90.

2 Yamagata K, Daitoku H, Takahashi Y, Namiki K, Hisatake K, Kako K et al. Arginine methylation of FOXO transcription factors inhibits their phosphorylation by Akt. Mol Cell 2008; 32: 221-31.

3 Cong L, Ran FA, Cox D, Lin S, Barretto R, Habib N et al. Multiplex genome engineering using CRISPR/Cas systems. Science 2013; 339: 819-23.
